# Design of an ultra-wideband omnidirectional and polarization insensitive flower petal antenna for potential ambient electromagnetic energy harvesting applications

**DOI:** 10.1038/s41598-022-09991-3

**Published:** 2022-04-12

**Authors:** Wei-Chih Wang, Prabir Garu

**Affiliations:** 1 Institute of NanoEngineering and MicroSystems, National Tsing Hua University, Hsinchu, 30013 Taiwan, ROC; 2 Department of Power Mechanical Engineering, National Tsing Hua University, Hsinchu, 30013 Taiwan, ROC; 3 Department of Electrical Engineering, University of Washington, Seattle Washington, 98195 USA; 4 Department of Mechanical Engineering, University of Washington, Seattle Washington, 98195 USA

**Keywords:** Mid-infrared photonics, Solar energy and photovoltaic technology, Electrical and electronic engineering, Terahertz optics, Green photonics, Devices for energy harvesting

## Abstract

Developing a polarization insensitive, omnidirectional, and ultra-wideband (UWB) antenna is highly desired for improving the utilization of freely available electromagnetic (EM) radiation energy. In this study, we have designed an UWB antenna based on tapered flower petals and numerically analyzed to show that it is a promising candidate for energy harvesting applications in the infrared (IR) to UV–visible regime. The impacts of design strategy and parameters on the absorption performance are studied numerically. The antenna shows a high performance in both bandwidth and absorptivity (average absorption of 84.5% spanning a broad range from 25 to 800 THz) under normal incidence of plane waves. To get a better understanding behind such high and UWB absorption mechanism, we investigated the electric field (E-field) distribution over the structure. The antenna also generates less than 5% absorption deviation between normal to 45° incident angle and 0.05% absorption deviation between 0° and 90° polarizations for both transverse electric (TE) and transverse magnetic (TM) modes. This new design aspect and the numerical findings unfolds the new direction for numerous EM wideband applications such as THz technology, photo detection, bolometric sensing, camouflaging, spectral imaging, and ambient EM energy harvesting applications.

## Introduction

The power is often the limiting factor, as the modern society is being highly dependent on battery sources for ultra-low power electronic devices. This leads to the tedious task of disposing and replacing large numbers of batteries that cause environmental pollution. Ambient EM energy harvesting (EH) offers a green and sustainable approach to this problem. This is a promising solution to provide a sustainable energy source to meet future demands of low power electronic systems. We are surrounded by EM energy in all frequency ranges. If we can capture and convert them into usable energy, we can use it to power many electronics that would help to address the energy challenges we are facing right now. To date, numerous approaches have been investigated for collecting such freely available EM radiation energy^1–17^. Among them, some of the research groups proposed and experimentally demonstrated mechanically flexible^1,7^, strongly polarization independent^2,6,14,16^, near unity^4,5,14^, and broadband absorbers^1,2,4–7,12,14,16^. However, the aforementioned propose structures can only work within a limited frequency band, which limits their practical applications. Karampour et al.^6^ and Alam et al.^9^ proposed and numerically investigated EM wave absorbers which can operate in the broadband IR region. Although, the structures show ultra-wideband and polarization-insensitive behavior, however their angular stability is quite low (incident angle $\uptheta \le 35$°), which causes practical limitations. Pala et al. experimentally investigated a prototype of angle insensitive, omnidirectional and broadband metasurface absorber based on two-dimensional periodic arrays of multi-resonant crossed trapezoidal Mie resonators^3^. At shorter wavelengths, the device can achieve a strong and broadband absorption, whereas at longer wavelengths, it exhibits quite low and narrowband absorption spectrum. Sun et al. proposed, fabricated, and experimentally demonstrated a cost effective wafer scale broadband THz absorber based on vertically aligned carbon nanotube (VACNT) arrays^8^. This is, however, challenging to obtain optimum parameters (volume fraction and alignment factor) practically due to the difficulties of controlling each parameters accurately throughout the fabrication process. Lv et al. demonstrated experimentally a broadband THz absorber based on engineered subwavelength surface relief grating structure^11^. Despite being polarization insensitive up to 60°, the structure suffers from uniform and smooth absorption spectrum and can only absorb EM waves in a narrow bandwidth over the THz regime. Yuan et al. have demonstrated a new type THz absorber based on antireflection techniques into doped silicon^13^. This structure can achieve high and broadband absorption, however, it shows some parametric deviations between measured results and simulated results due to the fabrication imperfection. The delicate differences in lithography process make deviations on such structure parameters. Zhu et al. numerically demonstrated a hybrid nano-antenna for IR and visible energy harvesting applications^15^. Although the proposed absorber can achieve strong and broadband absorption at normal incidence, however, it shows slightly weak absorption performance under different polarization angles. The average absorption decreases slightly with the increase in polarization angle due to structural asymmetry in some directions. Xiao et al.^7^, Haque et al.^10^, and Wu et al.^17^ also demonstrated ultra-wideband THz EM wave absorbers. Despite their strong and broadband performance, the structures suffer from fabrication difficulties in the lithography process due to their complicated nanostructure layout and several process steps. Therefore, a straightforward design strategy for new kind of structures is of great interest in order to overcome the limits of the aforementioned existing structures. Ultrathin wideband absorber with freely tunable operating frequencies across a wide frequency range, including the IR, visible, and UV–visible domains have yet to be investigated.

The concept of capturing EM energy using antenna has been around few decades, since late 1950s^18^. This involves collecting ambient EM radiation and convert it into electrical energy for powering the low power electronic devices. An energy harvester can do the job to convert such ambient EM energy into useable electrical energy. Bailey proposed the rectenna concept in the field of solar energy harvesting in the early 1970s, where rectenna stands for rectifying diode coupled with an antenna^19^. Solar energy is the most abundant and direct energy source consists of 52% IR, 43% visible and 5% UV–visible energy^20^. Among all the visible energy harvesting is primarily implemented on an industrial scale using photovoltaic (PV) and thermal solar cells. However, these cells suffer from performance due to certain factors such as narrowband absorption, bad weather and only daytime supply of visible light. Therefore, an alternative approach that can alleviate such problems is highly desirable. Moreover, the traditional solar cells are not collecting most of the solar radiation in the IR region. In order to deal with the current renewable energy crisis, collect waste heat at infrared wavelengths and even longer wavelengths and turn it into usable energy is highly desirable.

From a theoretical point of view, nothing restricts the construction of electronic devices capable of harvesting wavelengths shorter than radio frequencies (RF). A fraction of the energy carried by EM waves can be obtained if this oscillating signal can then be rectified into a direct flow of electronic charges. Rectifying antennas, often called rectennas, are devices suitable for such operation. They are composed of two main components: an antenna built to fit the size of the wavelength to be harvested, and a rectifier circuit working at the frequency of the radiation harvested. The antenna is used to convert the incident EM wave into an alternating current (AC) signal, whereas the diode helps to rectify the AC signal to the usable direct current (DC) signal. Within the past two decades, there has been a surge of interest in and the subject of intense research in the above research. Very few studies have attempted to develop a fully rectifying antenna device that works on the visible and IR frequency band. The efficiency of rectifying antennas is still restricted by several challenges arising from the miniaturization of the system according to the desired wavelength. Despite the difficulty of overcoming these limitations, rectifying antennas remain very attractive as their operating frequencies do not depend on a bandgap whereas their semiconductor counterparts do. Although there is still no strong consensus on the theoretical ultimate performance of such devices, research in this field is still inspired by promising applications for high performance solar cells as well as tunable photoreactors in the IR range^21,22^. In the radio frequencies, the conversion efficiency over 90% can be obtained by the rectenna. However, extending the rectenna to the high frequency regime (above RF) is somewhat challenging due to the indirect process and the much too slow response of the diode-based rectification^23,24^. Therefore, an alternative approach must be developed to overcome those aforementioned issues. In 2020, physicists at MIT came up with a blueprint for a device that would be able to convert ambient terahertz waves into a direct current. Isobe et al. first proposed that method which can directly convert the incoming EM radiation into DC current^25^. Based on their findings, we may claim that once we develop such system, our proposed antenna could also be used to convert the incoming EM energy (above IR) into usable DC electrical energy.

The concept of using non-resonant nano-antennas for the harvesting of EM energy has received a lot of attention, as they provide a way of collecting and locating the energy of free-propagating waves in limited volumes^26,27^. This nanostructures use the wave design of electromagnetic radiation to transfer optical energy to localized resonant currents that are ultimately used to retrieve confined energy, thereby opening a way for solar device engineering^28,29^. The successful integration of nano-antennas into photovoltaic technology is based on the implementation of an effective, retrieval mechanism, currently non-existent. Nano-antennas coupled to high-speed rectifiers (known as 'rectennas'), based on tunnel barriers, have been extensively investigated in recent years as optical harvesting devices in this context^30–32^. Despite the high theoretical efficacy they can obtain, rectennas experience low efficiencies owing to the poor efficiency of the existing rectifiers at optical frequencies^33–35^. Other harvesting mechanisms must be investigated as we wait for an efficient rectifier to be created. Herein, we have explored a new design concept of tapered flower structure which combines the use of nano-antenna to confine the IR, entire visible and part of UV–visible band. The proposed design comprises of metal-dielectric-metal (MDM) configuration in which the top metallic plate and sandwiched dielectric layer are tapered to generate a groove-like structure which enables the formation of analog structure to confine and cover the wavelength of different frequencies.

## Electromagnetic energy harvesting device

### Electromagnetic wave energy absorbers

Perfect EM wave absorbers are devices in which all incident radiation is absorbed efficiently at the operating wavelengths. Once the radiation is absorbed by the devices, it transformed into ohmic heat or other form of energies. Thus, reflection, transmission, scattering and all other waves propagation are not observed as they pass through the perfect absorbers. In general, conventional absorbers are made of materials with high intrinsic losses, but the EM absorbers that we are concerned with are mainly made of noble metals. Based on the absorption mechanism, EM absorbers can be classified into two categorize: resonant and broadband absorbers. Resonant absorbers rely on the material at a particular frequency ${\omega }_{0}$ (where the wavelength corresponding to ${\omega }_{0}$ is ${\lambda }_{0}=\frac{2\pi c}{{\omega }_{0}}$ and $c$ is the speed of light in vacuum) to interact with the incident radiation in a resonant manner. Due to the desired resonance of the material at a given wavelength, the resonant absorbers are frequency-dependent. Absorption reaches its maximum at the resonance frequency. Broadband absorbers on the other hand rely on materials whose properties are independent in frequency, and can therefore absorb radiation over a wide range of frequencies. The goal of the perfect broadband absorbers is to minimize reflection and transmission and maximize the absorption bandwidth in the broad frequency range. In broadband operation, although transmission can be easily blocked by simply placing a metallic plate, minimizing the reflection over a wide range of frequency is not trivial. Therefore, research on minimizing reflection of broadband absorbers has gained remarkable and increasing attention in recent decades.

Broadband absorbers, such as antennas, help to capture EM waves that can be used for low power electronic devices. However, EM radiation energy cannot be stored directly into battery or capacitors. Therefore, we need to convert the AC radiation power into DC electrical power if we want to use them in our practical applications. Rectennas are the ideal candidates that can play such a role to collect EM waves from a variety of sources and transform them into DC useful power. Another approach of strong magnetic resonant coupling which is a non-radiating technique was recently applied in highly efficient wireless power transfer technology^36^. Both the inventions discussed above are involved power transfer instead of power generation. Therefore, an external power source is required in order to emit the EM radiation.

When we talk about energy conversion mechanism of any devices, the conversion efficiency comes to play the major role for the device performance. Therefore, an efficiency equation is needed to know the performance of the device. The conversion efficiency is the quantitative expression of the device that combines energy input and energy output in terms of energy conversion. The total efficiency $({\eta }_{tot})$ of the energy conversion device is divided into three parts: the absorption efficiency (${\eta }_{a}$), conversion efficiency (${\eta }_{c}$), and loading efficiency (${\eta }_{l}$) related to impedance (Z) matching and can be written as

1 $${\eta }_{tot}={\eta }_{a}{\eta }_{c}{\eta }_{l}$$

The current technology is focused on nano-rectenna device to harvest the EM radiation efficiently. However, there is a big problem with the total harvesting efficiency of the rectenna over a broad frequency ranges. The amount of power that is lost as a result of the antenna and rectifier impedances never matching perfectly^26,33,34^. Therefore, several studies have attempted to harvest EM energy with 100% efficiency and some with extremely broadband operation using the energy generators in which the energy absorber is embedded on it^12,34,37^. The broadband and perfect absorbers that can achieve unity absorption over a wide frequency band make energy absorbers promising candidates for energy harvesting applications^38,39^. In the process of energy harvesting by these devices, two critical steps can be identified. The first step is ensuring that the absorber is absorbing the EM radiations. Step 2 is to relate to the electronic component, where an electronic circuit has to rectify the oscillating free charges induced by the absorbed radiations. The sizing or dimension of the electronic component is related to the frequency of the EM radiations it interacts with. In such a device, the absorption of EM waves is no longer dependent on the bandgap of a semiconductor material, but rather on the dimension of the metallic antenna exposed to radiations.

### Design, modeling, and simulation of the proposed antenna

**Figure 1:**
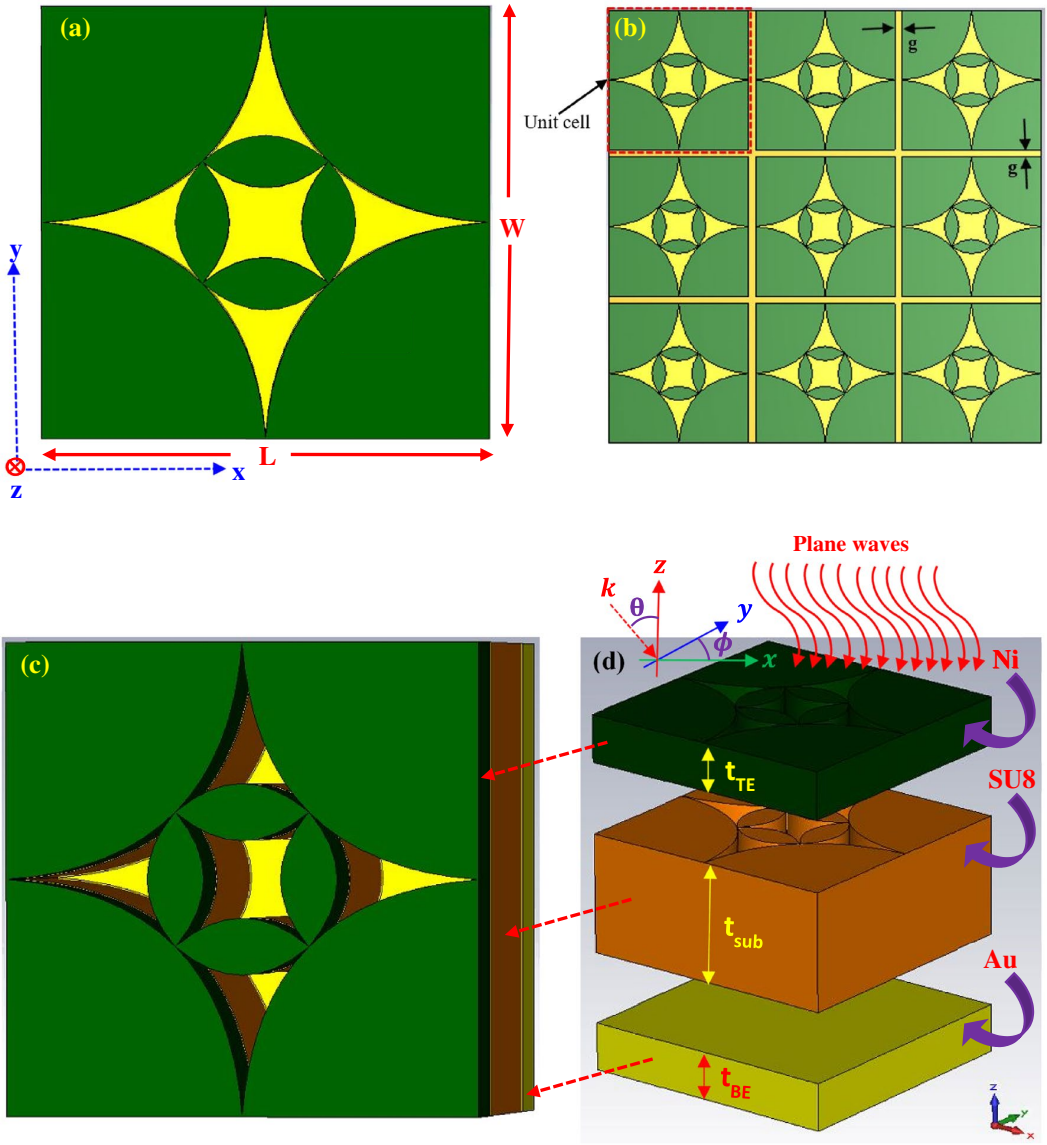
Schematic of the designed broadband antenna: (**a**) front view of the unit-cell structure and (**b**) 2-D periodic array (3 × 3 unit cells) structure in x–y plane. The unit-cell is composed of metallic (Ni) and dielectric (SU8) flower petals over the ground gold (Au) plate, (**c**) perspective view of the unit-cell (the top metal (Ni) and substrate (SU8) are patterned and shaped in a groove like structure), (**d**) 3D demonstration of the structure with a unit cell, in which each layer is separated from each other for clear observation.

**Figure 2:**
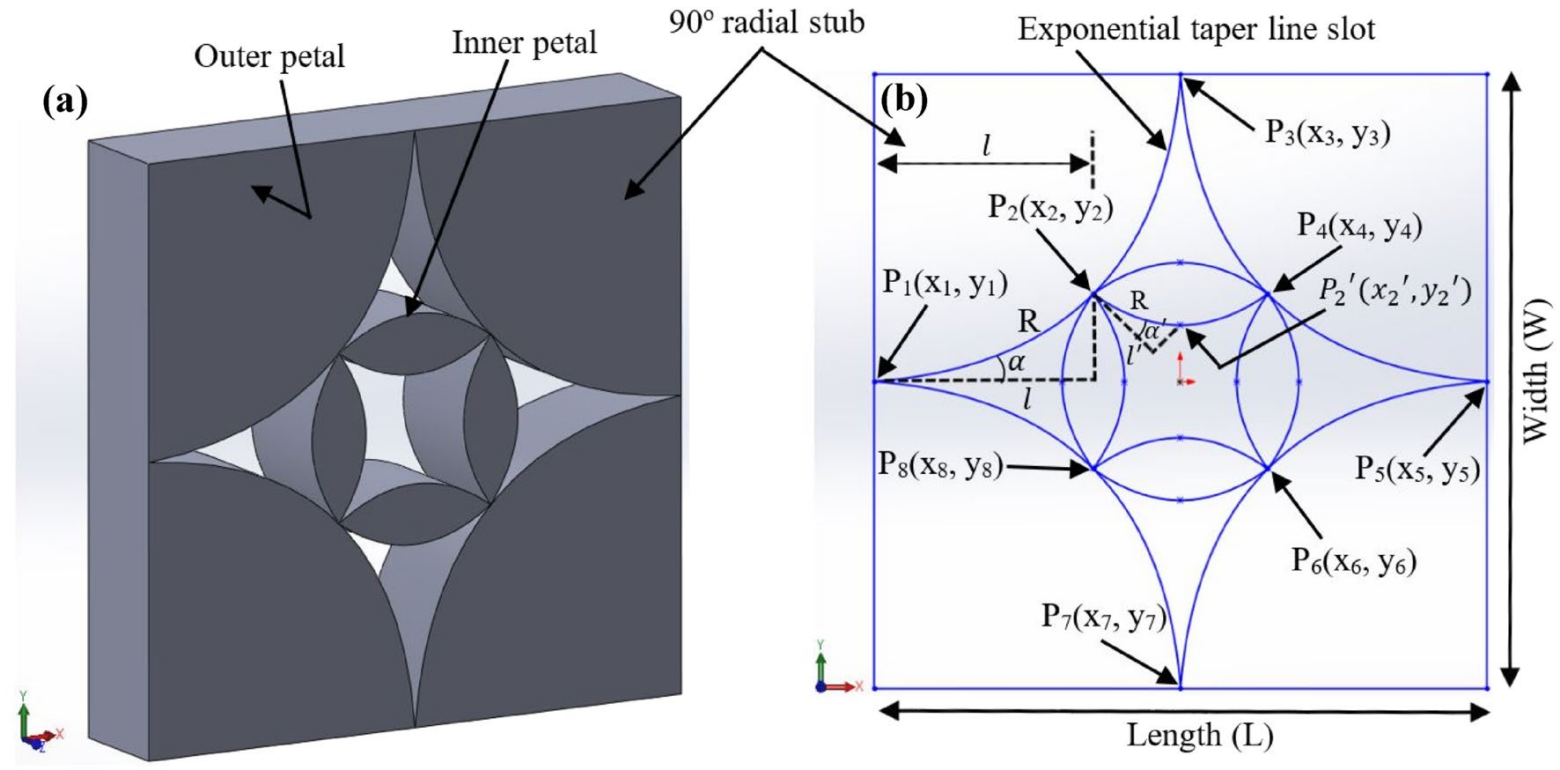
Schematic of the (**a**) perspective view and (**b**) planar top view (in the x–y plane) of the tapered antenna structure.

The illustration of the proposed UWB antenna is manifested in Fig. 1a–d. The top and perspective view of the unit cell of the proposed antenna are shown in Fig. 1a,c, respectively. A large 2-dimensional (2-D) periodic array of the unit cells (3 × 3 array) is depicted in Fig. 1b. Figure 1d displays the clear view of the unit cell structure in which all the layers are separated from each other. The structure consists of a top layer of lossy metal nickel (Ni), a dielectric SU8 layer and a bottom layer of lossy metal gold (Au). The top metal and middle dielectric layers are patterned whereas the bottom Au plate was not pattern, instead it was kept continuous layer to avoid transmission of EM waves. The dielectric SU8 layer was sandwiched between the top and bottom metal electrode plates. Due to the thermal properties and thickness compatible with the proposed design, SU8 was selected for the dielectric substrate^40^. Properties of the materials used in the simulation model are listed in Table 1^41–60^. Thickness of the top and bottom metal electrode layers were 200 nm. The substrate thickness was 500 nm with a relative permittivity (${\varepsilon }_{r}$) of 2.8. The overall thickness of the proposed unit cell antenna is 900 nm. For the periodic array structure, the gap (g) between unit cells is 100 nm. The geometric parameters and dimensions of proposed antenna are summarized in Table 2. The top metal and middle dielectric layers of the structure are tapered in such a way that generate four identical flower petals near the center of the structure (inner petals) and four identical flower petals outside of the inner petals of the structure (outer petals). Each inner petal near the center of the structure is designed using six-spline curve in SOLIDWORKS® and then the resulting structure is rotated by 90° to obtain the symmetric structural arrangement of desired inner flower petals. On the other hand, each outer petal is designed by using three-spline curve and the resulting structure is rotated by 90° to obtain the symmetric structural arrangement of desired outer flower petals. Such tapered design is a more analog approach of covering the wavelength of different frequencies results in flat/smooth wideband absorption. Due to the symmetry of the structure, it allows polarization not to be a factor in the detection of plane waves. The generated inner petals cover the higher frequency regime whereas the outer petals cover the lower frequency regime for the absorption of EM waves. The unit cell of the proposed antenna was split into three sections in order to clearly observe the structure, as shown in Fig. 1d. The pattern SU8 layer was stacked over the bottom electrode and it did not cover the entire area of the electrode (the bottom layer was only covered by its flower petals region). Antenna taper is defined as exponential curves in the x–y plane as shown in Fig. 2a,b. For the outer petals, the exponential taper profile is defined by the opening rate R and two points ${P}_{1}\left({x}_{1}, {y}_{1}\right)$ and ${P}_{2}\left({x}_{2}, {y}_{2}\right)$ ^61^

**Table 1:** Properties of the materials used for the numerical model.

Properties	Materials		
	Gold (Au)	Nickel (Ni)	SU8
Relative permittivity (${\varepsilon }_{r}$)^41^	–	–	2.8
Relative permeability (${\mu }_{r}$)^42–44^	1	600	1
Electrical conductivity ($\sigma$) [S/m]^45,46^	4.10e + 07	1.64e + 07	–
Thermal conductivity (k) [W/k/m]^47–49^	314	91	0.2
Density ($\rho$) [kg/m^3^]^50–52^	19,320	8900	1200
Heat capacity (${C}_{p}$) [kJ/K/kg]^53,54^	0.13	0.45	1.6
Young modulus (Y) [kN/mm^2^]^55–57^	78	207	4.02
Poisson’s ratio (P)^48,54^	0.42	0.31	0.22
Thermal expansion coefficient ($\alpha$) [1e − 6/K]^58–60^	14.1	13.3	87.1

**Table 2:** The geometric parameters and dimensions of proposed antenna (nm).

Design name	Unit cell length (L)	Unit cell width (W)	Substrate thickness (t_SUB_)	Top and bottom metal thickness (t_TE/BE_)	Unit cell thickness
Flower petal	1000	1000	500	200	900

2 $$y={c}_{1}{e}^{Rx}+{c}_{2}$$

where,

3 $${c}_{1}=\frac{{y}_{2}-{y}_{1}}{{e}^{R{x}_{2}}-{e}^{R{x}_{1}}}$$

4 $${c}_{2}=\frac{{y}_{1}{e}^{R{x}_{2}}-{y}_{2}{e}^{R{x}_{1}}}{{e}^{R{x}_{2}}-{e}^{R{x}_{1}}}$$

The outer taper length $l$ is ${x}_{2}-{x}_{1}$. For the taper design, the opening rare R varies from 0 to 0.7. In the limiting case where R approaches zero, the exponential taper results in a so-called linearly tapered slot antenna for which the outer taper slope is constant and given by

5 $${S}_{o}=\frac{{y}_{2}-{y}_{1}}{{x}_{2}-{x}_{1}}$$

For the exponential taper defined by Eq. (2), the outer taper slope, S changes continuously from S_1_ to S_2_, where S_1_ and S_2_ are the outer taper slopes at ${x=x}_{1}$ and at ${x=x}_{2}$, respectively, and S_1_ < S < S_2_ for R > 0. The outer taper flare angle is defined by $\alpha ={tan}^{-1}S$.

In the same way, for the inner petals, the exponential taper profile is defined by the opening rate R and two points ${P}_{2}\left({x}_{2}, {y}_{2}\right)$ and ${{P}_{2}}^{^{\prime}}({{x}_{2}}^{^{\prime}}, {{y}_{2}}^{^{\prime}})$

6 $${y}^{^{\prime}}={{c}_{1}}^{^{\prime}}{e}^{Rx}+{{c}_{2}}^{^{\prime}}$$

where,

7 $${{c}_{1}}^{^{\prime}}=\frac{{{y}_{2}}^{^{\prime}}-{y}_{2}}{{e}^{R{{x}_{2}}^{^{\prime}}}-{e}^{R{x}_{2}}}$$

8 $${{c}_{2}}^{^{\prime}}=\frac{{y}_{1}{e}^{R{{x}_{2}}^{^{\prime}}}-{{y}_{2}}^{^{\prime}}{e}^{R{x}_{2}}}{{e}^{R{{x}_{2}}^{^{\prime}}}-{e}^{R{x}_{2}}}$$

The inner taper length ${l}^{^{\prime}}$ is ${{x}_{2}}^{^{\prime}}-{x}_{2}$. In the limiting case where r approaches zero, the exponential taper results in a so-called linearly tapered slot antenna for which the inner taper slope is constant and given by

9 $${{S}_{0}}^{^{\prime}}=\frac{{{y}_{2}}^{^{\prime}}-{y}_{2}}{{{x}_{2}}^{^{\prime}}-{x}_{2}}$$

For the exponential taper defined by Eq. (6), the inner taper slope, ${S}^{^{\prime}}$ changes continuously from ${{S}_{1}}^{^{\prime}}$ to ${{S}_{2}}^{^{\prime}}$, where ${{S}_{1}}^{^{\prime}}$ and ${{S}_{2}}^{^{\prime}}$ are the inner taper slopes at ${x=x}_{2}$ and at $x={{x}_{2}}^{^{\prime}}$, respectively, and ${{S}_{1}}^{^{\prime}}$ < ${S}^{^{\prime}}\hspace{0.17em}<\hspace{0.17em}{{S}_{2}}^{^{\prime}}$ for R > 0. The inner taper flare angle is defined by ${\alpha }^{^{\prime}}={tan}^{-1}{S}^{^{\prime}}$. Rest of the inner and outer tapered petals are designed using other coordinates ${P}_{3}\left({x}_{3}, {y}_{3}\right)$, ${P}_{4}\left({x}_{4}, {y}_{4}\right)$,${P}_{5}\left({x}_{5}, {y}_{5}\right)$, ${P}_{6}\left({x}_{6}, {y}_{6}\right)$, ${P}_{7}\left({x}_{7}, {y}_{7}\right)$, ${P}_{8}\left({x}_{8}, {y}_{8}\right)$. The antenna taper design parameters and dimensions are listed in Table 3.

**Table 3:** Design parameters and dimensions of the proposed tapered structure.

Parameters	Dimensions
$l$	360 nm
${l}^{^{\prime}}$	140 nm
$\alpha$	${10.7}^{o}$
${\alpha }^{^{\prime}}$	${15.8}^{o}$
$S$	0.18
${S}^{^{\prime}}$	0.28

The selection of appropriate materials was essential prior to the design of the EM energy absorber antenna. Since the materials properties are incomplete in the frequency band of operation, Drude free-electron model is considered for modeling the complex permittivity of the metals, instead of combining conductivity and real permittivity^62^. According to the model the complex relative dielectric function can be expressed as^63^:

10 $${\varepsilon }_{r}(\omega )={\varepsilon }_{r1}(\omega )+{i\varepsilon }_{r2}(\omega )=1-\frac{{{\omega }_{p}}^{2}}{{\omega }^{2}+i\omega \Gamma }$$

where, $\omega$ is the external EM radiation frequency, $\Gamma$ is the damping frequency, ${\omega }_{p}$ is the plasma frequency defined by ${{\omega }_{p}}^{2}=\frac{N{e}^{2}}{{m}^{*}{\varepsilon }_{0}}$, where, N is the free electron concentration in metal, ${\varepsilon }_{0}$ is the free space permittivity, e and ${m}^{*}$ are the electronic charge and effective mass, respectively. The first term in Eq. (10), i.e., ${\varepsilon }_{r1}(\omega )$ is the dielectric constant contributed by valence electrons (bound electrons). At very high frequencies above electronic absorption, the value of ${\varepsilon }_{r1}(\omega )$ is one. On the other hand, the second term in Eq. (10) arises from the free electrons. In the Drude model, the complex conductivity of the metal can be expressed as^64^:

11 $$\sigma \left(\omega \right)={\sigma }_{r}\left(\omega \right)+i{\sigma }_{i}\left(\omega \right)= \frac{i{\varepsilon }_{0}{{\omega }_{p}}^{2}}{\omega +i\Gamma }$$

where, ${\sigma }_{r}\left(\omega \right)$ and ${\sigma }_{i}\left(\omega \right)$ are the real and imaginary part of the conductivity, respectively, ${\varepsilon }_{0}=8.854\times {10}^{-12}(\mathrm{F}/\mathrm{m})$ is the free space permittivity.

The proposed unit cell antenna was modelled and simulated in the commercially available simulation software CST Microwave Studio (MWS) Suite. CST can solve Maxwell’s equations and ensure the reliability and accuracy of numerical results. The absorptivity of the antenna was calculated and optimized numerically based on the Finite Element Method (FEM) in the frequency domain. In order to calculate the absorptivity of the antenna, it is necessary to obtain the complex frequency dependent S-parameters, ${S}_{11}(\omega )$ and ${S}_{21}(\omega )$. Therefore, auto meshing and frequency-domain solver (FDS) were used in the simulation to obtain S-parameters. In simulation, periodic boundary conditions (PBCs) were applied in the x-axis and y-axis, and open boundary condition (OBC) was used along z-axis. The frequency domain solver was used with a plane wave source that propagates in the negative z direction to analyze the electromagnetic characterization over the unit cell antenna. In order to describe the EM features of the proposed design individually, the EM energy absorption spectrums are derived from the simulation. Considering EM energy conservation, the summation of absorbance, reflectance, and transmittance is equal to one. The normalized spectral absorbance (A) of the antenna can therefore be obtained by

12 $$A=1-R\left(\omega \right)-T\left(\omega \right)=1-{\left|{S}_{11}\right|}^{2}-{\left|{S}_{21}\right|}^{2}$$

where $R\left(\omega \right)={\left|{S}_{11}\right|}^{2}$ and T $\left(\omega \right)={\left|{S}_{21}\right|}^{2}$ are the reflectance and transmittance, respectively, retrieved from the frequency dependent S-parameters. The average absorptance was calculated by

13 $$A={\int }_{{\lambda }_{1}}^{{\lambda }_{2}}\frac{A\left(\lambda \right)d\lambda }{({\lambda }_{1}-{\lambda }_{2})}$$

where, ${\lambda }_{1}$ and ${\lambda }_{2}$ are the wavelengths of the incoming plane waves corresponds to the frequencies of 800 THz and 25 THz, respectively.

## Electromagnetic characterization results

### Absorption, impedance, power dissipations, E-field distribution, and radiation patterns

**Figure 3:**
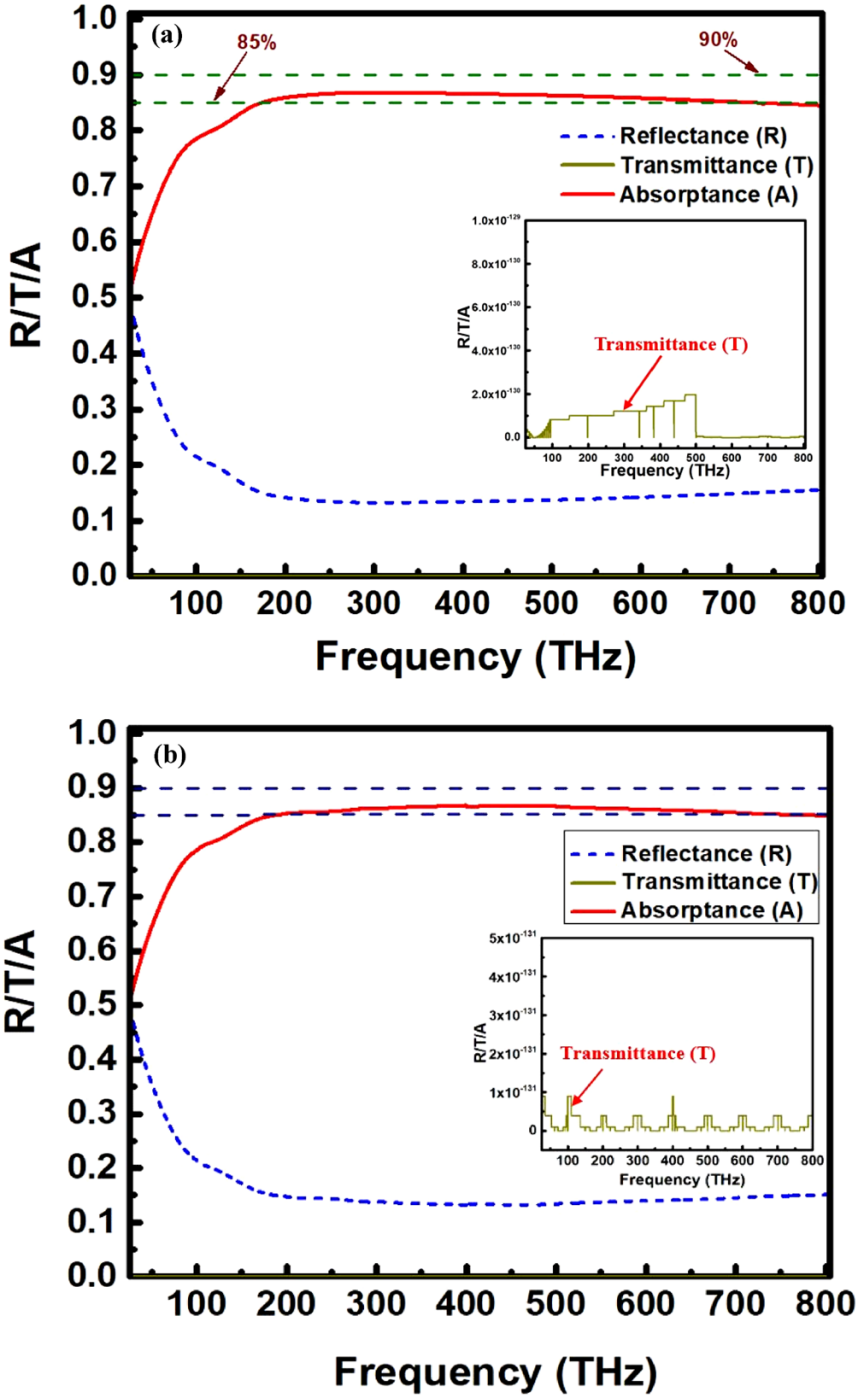
Reflectance, transmittance and absorption spectrums of proposed (**a**) unit-cell and (**b**) 2-D periodic array (3 × 3 unit cells) broadband antenna under normal incidence of plane wave: The top metal (Ni) and substrate (SU8) are patterned and shaped in a groove like structure. The inset images are the magnified view of the transmittance spectrum.

To shed light on the physical origin of ultra-wideband absorption of the proposed unit cell and 2-D periodic array antenna, we simulated the structures under normal incidence to obtain absorptance, reflectance and transmittance spectra depicted in Fig. 3a,b. The unit cell antenna has an excellent absorption bandwidth of 84.5% absorbance reaches as high as 775 THz from 25 to 800 THz. The absorption performance of the periodic array antenna is not so differing from the unit cell antenna, follows almost the similar trend to that of unit cell antenna. The array antenna can obtain an average absorption of 83.4% within the operating frequency band. This UWB and high absorption originates from the mutual coupling and overlapping between the consecutive resonances in the unit cell structure. The interaction between the incoming waves and well-designed tapered flower petal structure cause more energy to be consumed by the antenna. In this case, most of the reflected waves are destructively coherent with each other within the operating frequency range, and therefore less amount incident wave is reflected back from the surface, resulting in a wide band absorption.

We set up a periodic structure with boundary conditions and the incident waves that are excited along the z-axis by using Floquet ports at the top and bottom of the structure. The incident wave will be reflected or absorbed as it propagates as a plane wave on this structure, depending on the mismatch of impedance of the structure to the free space impedance. The strong impedance matching between the antenna and the surrounding free space might be another possible reason to achieve this broadband characteristic. The bottom metal layer of the proposed antenna is sufficiently larger than the skin depth in the operating frequency regime, which causes negligible transmission (nearly zero) and results in high structural absorption. According to an analysis of the theory of electromagnetic wave absorption, the absorption of EM radiation by the absorber can be expressed as^65^

14 $$A=1-R=1-{\left|\frac{Z-{Z}_{0}}{Z+{Z}_{0}}\right|}^{2}=1-{\left|\frac{{Z}_{r}-1}{{Z}_{r}+1}\right|}^{2}$$

where, ${Z}_{r}=\frac{Z}{{Z}_{o}}$ is the relative impedance of the absorber, that can be extracted from the frequency dependent S-parameters,

15 $${Z}_{r}=\sqrt{\frac{{\left(1-{S}_{11}\right)}^{2}-{{S}_{21}}^{2}}{{\left(1+{S}_{11}\right)}^{2}-{{S}_{21}}^{2}}}$$

Since, the transmission through the device is almost negligible, Eq. (15) becomes

16 $${ Z}_{r}= \frac{1-{S}_{11}}{1+{S}_{11}}$$

**Figure 4:**
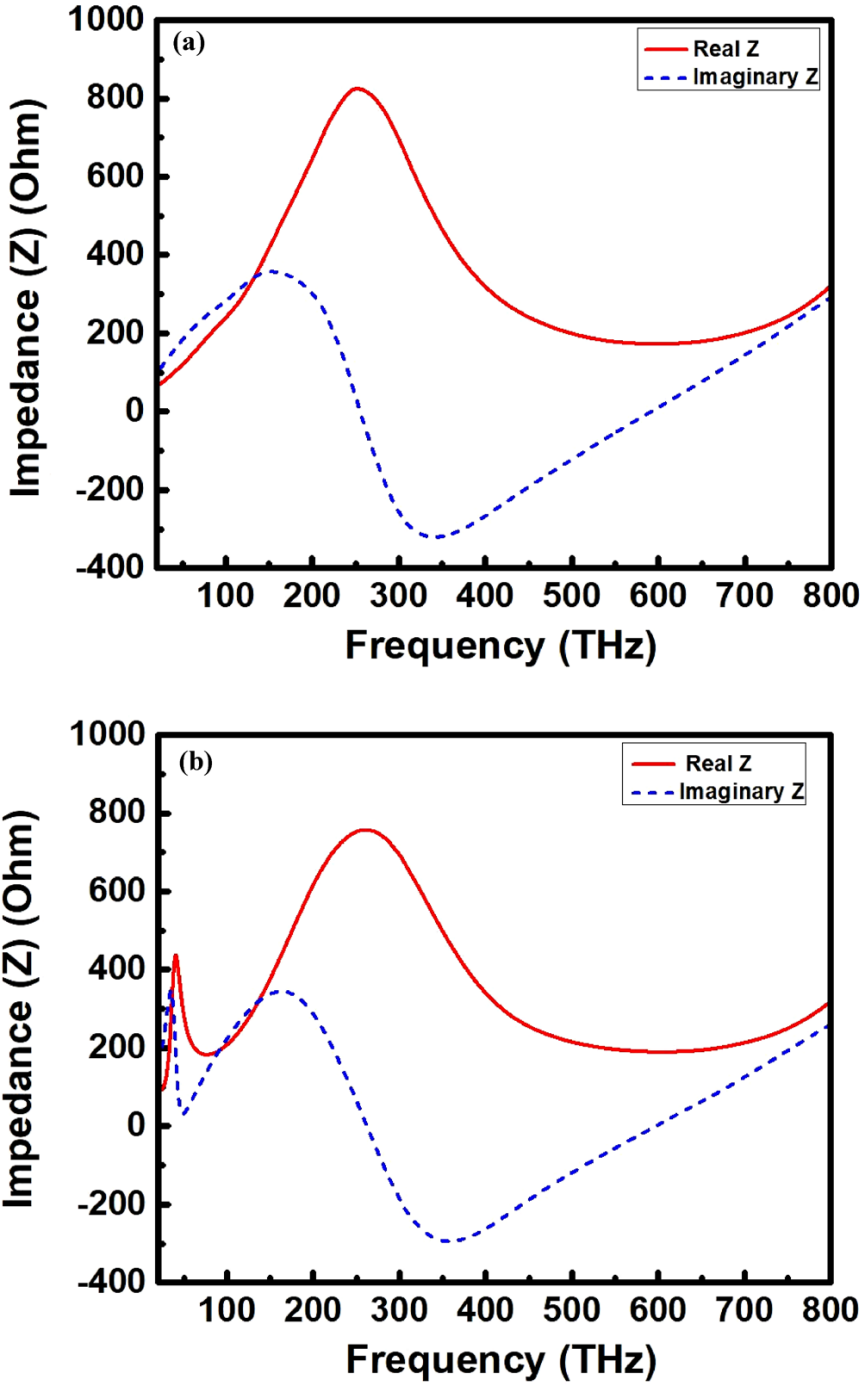
The real and imaginary parts of the impedance for (**a**) unit cell structure and (**b**) 3 × 3 array structure.

One conclusion can be drawn from Eq. (14) that more the relative impedance of the absorber matches the free space, the higher the absorption rate of the absorber. Figure 4a,b depict the real and imaginary parts of the impedance for the unit cell structure and 3 × 3 array structure, respectively. Some of the more closely related designs are referenced in Table 4 and their key features are summarized and compared with our proposed structure. It is also important to know the plasmonic behavior of the metal to figure out the underlying physics behind such UWB absorption at high frequencies. Generally, the broadband and high absorption mechanism at radio frequencies (RF) is quite straightforward. At these frequencies, the metals behave like perfect conductors with a skin depth becomes negligible compared to the size of the antenna. However, the wide band mechanism and high EM wave absorption above THz region is quite distinct from the radio frequencies. Metals, however, are not perfect conductors above THz frequencies, and are strongly correlated with plasmas^66^. The electrons in metals have significant inertia at these frequencies and cannot react instantaneously. The average skin depth is therefore in the order of tens of nanometers, comparable to the dimensions of the antenna. The geometry of the antenna thus scales with an effective wavelength that differs from the incident light wavelength. At this frequencies, the skin depth is appreciable and metals behave like free electron gas or plasma that are strongly coupled to incident light^67^. For most metals, free electrons have finite response time, above which they will not be fast enough to shield the bulk metal from incident fields. The inverse of this response time which is known as the plasma frequency. The typical value of this plasma frequency is around 500–1000 THz, correspond to electromagnetic waves in the visible and UV range. Metals are plasmonic in the regime near and just below the plasma frequency. Consequently, tiny metal objects will support resonances of the free electron gas that strongly interact with light. Successive resultant overlapping of these resonances results in high and ultra-wideband absorption by the structure. What actually happens for high absorption is, when high frequency (IR, visible or UV visible) EM waves imping on the metal surface, the real part of the dielectric constant of the metal becomes negative. Under the action of the E-field, the conduction electrons start to oscillate on the metal surface. When the oscillation frequency of the conduction electrons is consistent with the frequency of the incoming EM waves, the resonance is generated and surface plasmon polaritons (SPPs) occur. As a result, high absorption by the structure is observed, since, the structure based on SPPs has strong absorption capacity for high frequency EM waves. Therefore, it can be concluded that the structure is the promising candidate for the energy harvesting application in the high frequency band above mid infrared (MIR) region.

**Table 4:** Comparative study of different parameters of the proposed antenna with other absorbers.

Absorber name	Unit cell area $(\mathrm{\mu m}\times \mathrm{\mu m})$	Operating frequency range (THz)	Bandwidth (THz)	Average absorption (%)
Nanowires array absorber^1^	–	330–750	420	85
Plasmonic super absorber^2^	1.2 × 1.2	428–750	322	71
Mie resonators based metasurface absorber^3^	–	300–750	205 (545–750 THz)	> 80
Tunable plasmonic Equilateral triangular dielectric resonator nantenna (TP-ETDRN)^4^	5.0 × 5.0	185.1–194.7	9.6	~ 100
T-shaped nano-antenna^5^	–	1.0–5.0	4.0	~ 100
UT-shaped metamaterial absorber^6^	–	150–300	150	> 90
Vertically aligned carbon nanotube (VACNT) based absorber^7^	–	0.1–3.0	2.9	~ 98
Wafer scale VACNT based THz absorber^8^	–	0.3–2.5	2.2	> 98
Square- and rectangular-shaped patch absorber^9^	1.01 × 1.01	220–360	140	> 90
Bow-Tie Nano antenna^10^	18 × 18	23.2–27 and 31–35.9	3.8 and 4.9	~ 100
Surface relief grating structure^11^	21 × 14	3–5.1	2.1	> 90
Fermat spiral^12^	7.2 × 7.2	15–40	12.23	> 60
Grating structure^13^	100 × 100	0.44–10	9.56	> 90
Nano-antenna assisted solar absorber^14^	2.5 × 2.5	104.5–640.6	536.1	> 90
Hybrid nano-antenna^15^	1.1 × 1.1	166.5–749.5	583	> 84.5
Gradient nest ring-resonators^16^	178.2 × 178.2	2.57–5.19	2.62	> 90
FSA absorber^17^	0.3 × 0.3	200–750	550	~ 97.8
Flower petal (this work)	$1.0\times 1.0$	25–800	775	> 84.5

The structural components of the tapered antenna have also great influence on the UWB response. The nature of resonances of the inner and outer petals might be involved in the UWB absorption. The dependency of the resonant frequencies of different modes could be negligible on the periods between the inner and outer petals, which is indicative of localized modes instead of propagating modes such as surface plasmon polaritons (SPPs) or waveguide modes. These features support that the modes contributing to the UWB absorption are localized magnetic modes. The absorption spectrum is greatly extended by simply introducing tapers gap between both the inner and outer petals of the structure. This confirms the important role of localized gap SPPs in the tapered gaps on the enhanced absorption in the wide frequency ranges from 25 to 800 THz.

**Figure 5:**
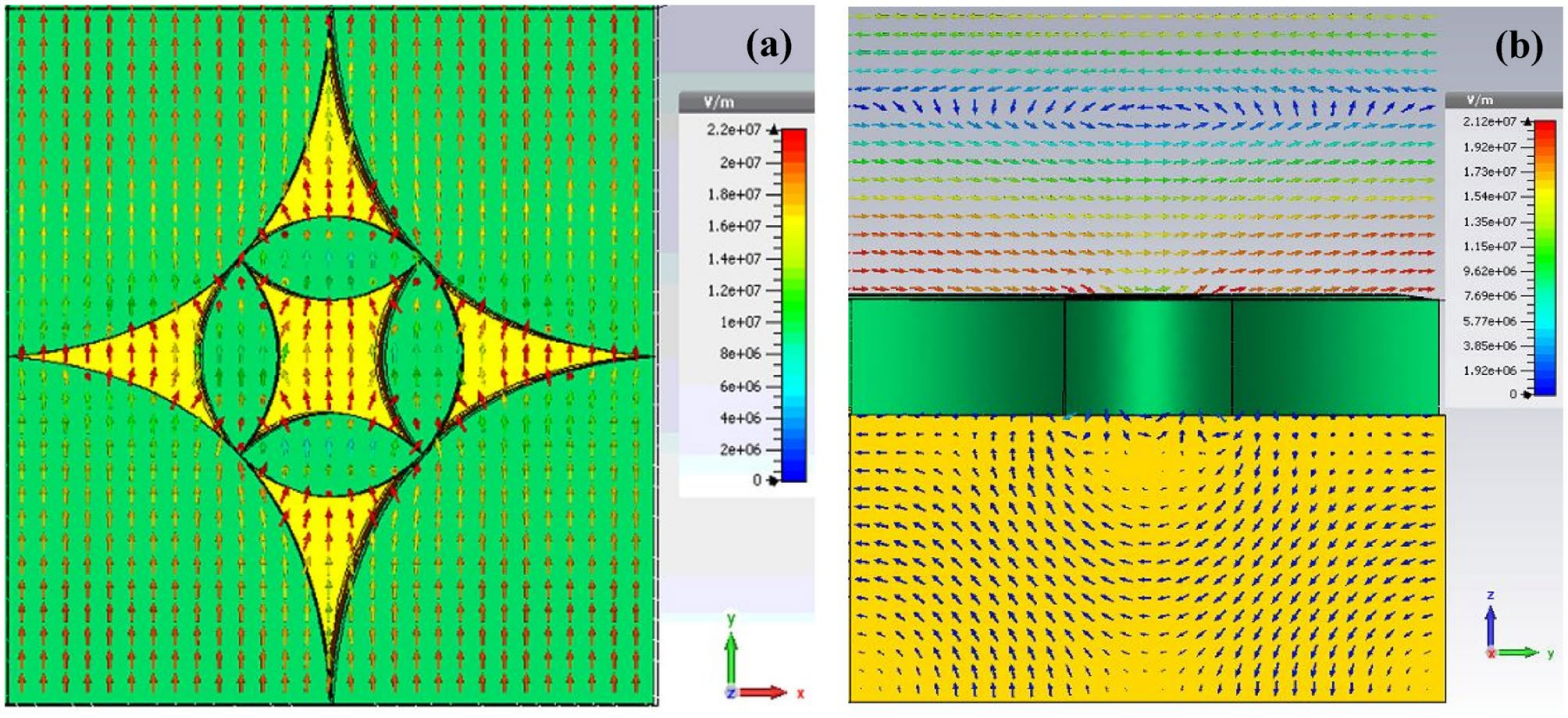
(**a**) Top view (**b**) cross-sectional view (at x = 0.141 µm) of electric field distribution of the proposed unit-cell antenna at 278.5 THz.

To figure out the underlying physics behind such ultra-wideband absorption, we further investigated the E-field distributions. One particular frequency 278.5 THz is selected to observe the top view (at z = 0.9 μm) in x–y plane and cross-sectional (at x = 0.141 μm) view in y–z plane of E-field distributions, as shown in Fig. 5a,b, respectively. It is observed that most of the E-fields are confined to the surface of both the outer and inner flower petals. The E-fields are stronger at the surface of both the inner and outer flower petals, which means that there is mutual influence between adjacent flower petals. It is also seen that the E-fields decrease significantly as EM waves propagating inside the absorber and almost vanish when reaching at the bottom Au plate (not shown here). This is in coincidence with the broadband absorption found in the absorption spectrum. In summary, the UWB absorption of the antenna is suggested to the consecutive electromagnetic resonance of the metal phase Ni generated at the multi-frequency point (from 170 to 800 THz), resulting in the efficient UWB absorption in the operating frequency regime.

**Figure 6:**
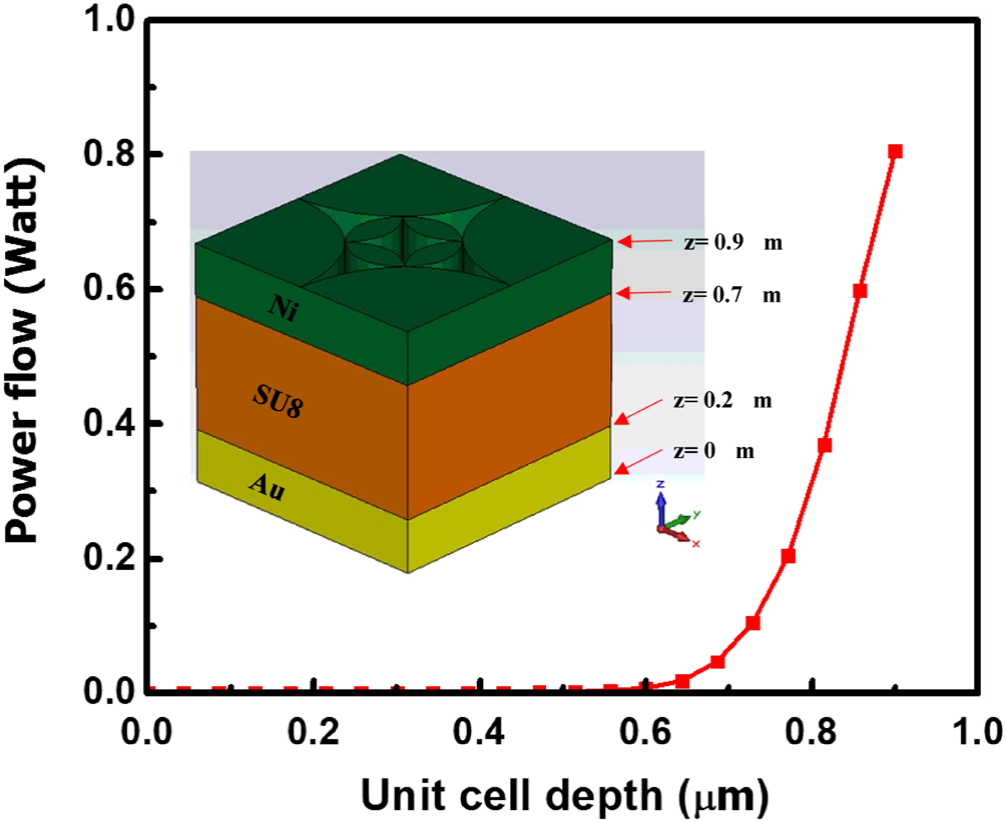
Power flow across different depth of the proposed unit-cell antenna at 278.5 THz.

Besides, absorption depth of the unit cell was also investigated, as shown in Fig. 6. The excitation power we used for the simulation was 1.0 W. From the figure, it is clear that significant amount of power (0.8 W at the surface) is absorbed by the top metal layer, and very less amount of power is absorbed by the sandwiched dielectric and bottom metal layers. The absorption is mainly attributed to the ohmic loss or resistive loss at the metal layer. Most of the absorption occurs on the support top metallic layer and can be transformed to temperature rise of the top metal film. In this structure, the power loss comes from the resistance loss ($q$), which can be expressed by^68^,

17 $$q=\frac{1}{2}{\varepsilon }_{o}\omega {\varepsilon }_{m}^{"}{\left|E\right|}^{2}$$

where, ${\varepsilon }_{o}$ is the dielectric constant of vacuum, $\omega$ is the angular frequency of the incoming wave,${\varepsilon }_{m}^{"}$ is the imaginary part of the relative permittivity of metal (${\varepsilon }_{m}={\varepsilon }_{m}^{^{\prime}}+{\varepsilon }_{m}^{"}$), E is the localized E-field intensity. Thus, in order to increase the absorption at non-resonant wavelengths, the dominating factor is to employ metals with high ${\varepsilon }_{m}^{"}$ to enhance the field dissipation. The above-mentioned outcome attributes that the structure with tapered flower petal configuration is the ideal candidate for UWB antenna design, which can be used for the wide band applications in the desired frequency ranges.

**Figure 7:**
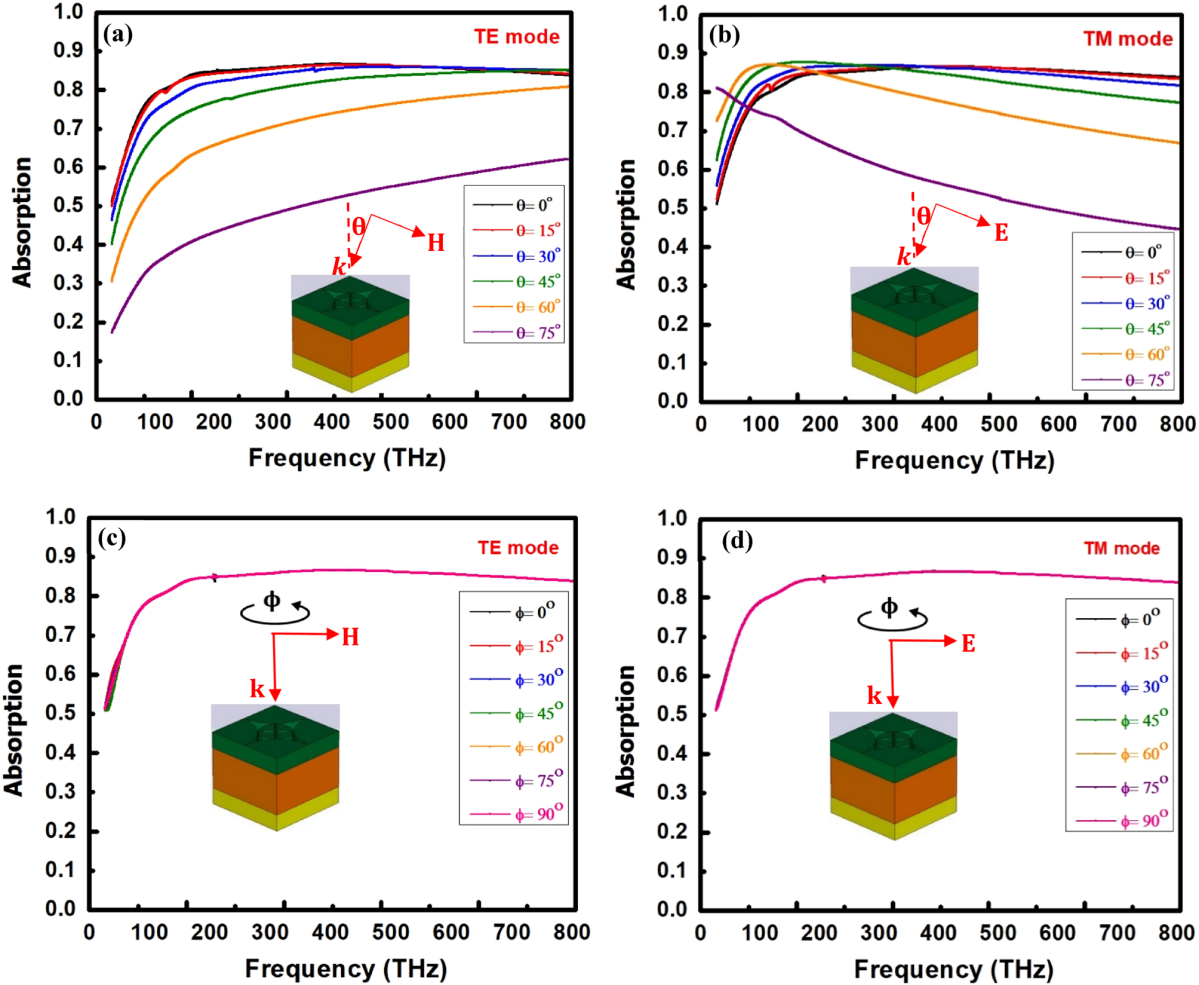
Simulated absorption spectra of the proposed antenna with different incidence angles θ ranging from ${0}^{o}$ to ${75}^{o}$ for (**a**) TE mode and (**b**) TM mode. TE and TM incident waves impinge on the absorber structure in x–z incident plane. Simulated absorption spectra of the proposed antenna for polarization angles ϕ ranging from ${0}^{o}$ to ${90}^{o}$ for (**c**) TE mode and (**d**) TM mode.

For practical applications, polarization-independent performance and a wide-angle incident wave are vitally important since, in some situations the incident wave is obliquely incident to the device. Therefore, polarization insensitivity of the proposed antenna was tested for normal as well as oblique incidences. Figure 7a,b show the absorption spectra of the proposed unit cell antenna under different incident angles (θ varies) up to 75° in the tread of 15° for both TE and TM mode polarizations. From the figures, it is seen that the antenna has no apparent change of absorption performance over the operating frequency ranges, maintained the average absorption above 82.7% for TE and 83.5% for TM mode under oblique incidence of incoming waves up to 45°. However, the absorption performance degrades above 45° for both mode of polarizations, which might be due to reflection and interference of the incoming waves. This finding indicates that the structure is polarization insensitive up to 45° for both the TE and TM modes. The unique mechanism of coupling between relevant electric and magnetic resonances and free-space incident light is attributed with the angle-independent absorption. In addition, to demonstrate the polarization insensitivity under normal incidence ($\theta =0$°) of plane waves, we also investigated the influence of the different polarization angles of the incident waves $\upphi$ up to 90° on the absorption performance for both TE and TM modes, as shown in Fig. 7c,d. From the figures, it is obvious that the average absorption is unchanged and maintained above 83.91% for TE and 83.92% for TM mode up to 90° under normal incidence of plane waves. This outcome attributes that the structure is strongly polarization insensitive up to 90° under normal incidence of incoming waves for both TE and TM modes. From the above findings, it can be concluded that at $\upphi =90$°, TE becomes TM and vice versa. The polarization insensitive nature of the structure is mainly due to the symmetrical arrangements of the inner and outer flower petal structures. Based on the above numerical results, it is obvious that the proposed unit cell antenna is polarization independent under both normal and oblique incidence of plane waves and maintained the absorption performance for both the TE and TM modes within the operation frequency band. Tables 5 and 6 display the average absorption for various incident angles ($\theta$ and $\upphi$) for TE and TM mode polarizations.

**Table 5:** Average absorption for different incident angle ($\theta$) for TE-mode and TM-mode polarization.

Various incident angle ($\theta$) in degrees	Average absorption for TE and TM mode	
	TE-mode absorption (%)	TM-mode absorption (%)
$\theta ={0}^{o}$	83.94	83.96
$\theta ={15}^{o}$	83.83	84.09
$\theta =3{0}^{o}$	82.99	83.93
$\theta ={45}^{o}$	80.32	82.39
$\theta ={60}^{o}$	72.33	75.95
$\theta ={75}^{o}$	51.58	56.37

**Table 6:** Average absorption for different incident angle ($\upphi$) for TE-mode and TM-mode polarization.

Various polarization angle ($\upphi$) in degrees	Average absorption for TE and TM mode	
	TE-mode absorption (%)	TM-mode absorption (%)
$\upphi ={0}^{o}$	83.91	83.93
$\upphi ={15}^{o}$	83.98	83.92
$\upphi =3{0}^{o}$	83.94	83.93
$\upphi ={45}^{o}$	83.87	83.93
$\upphi ={60}^{o}$	83.91	83.91
$\upphi ={75}^{o}$	83.92	83.92
$\upphi ={90}^{o}$	83.93	83.91

**Figure 8:**
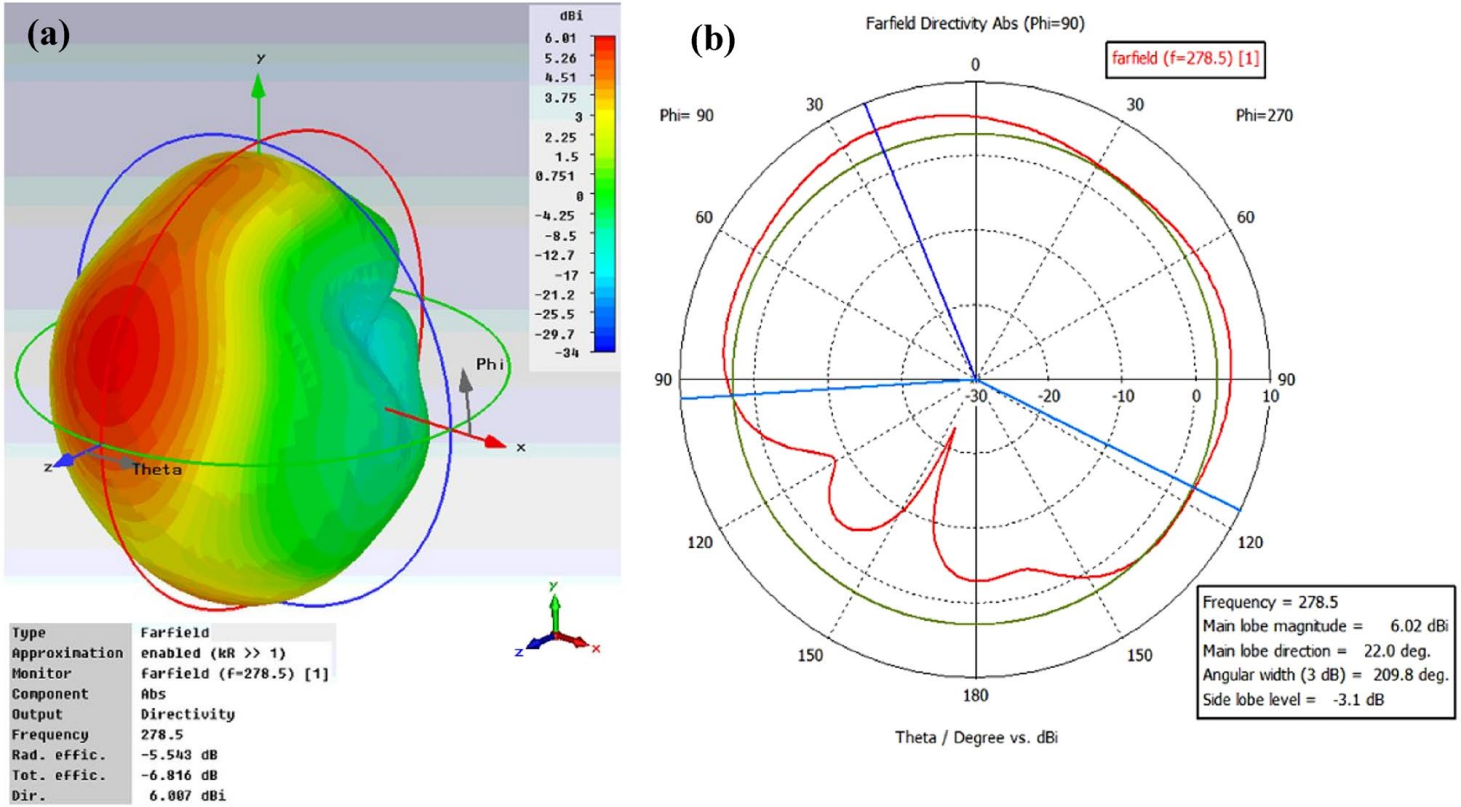
(**a**) 3D and (**b**) polar plot of farfield directivity of the proposed unit-cell broadband antenna at 278.5 THz.

**Figure 9:**
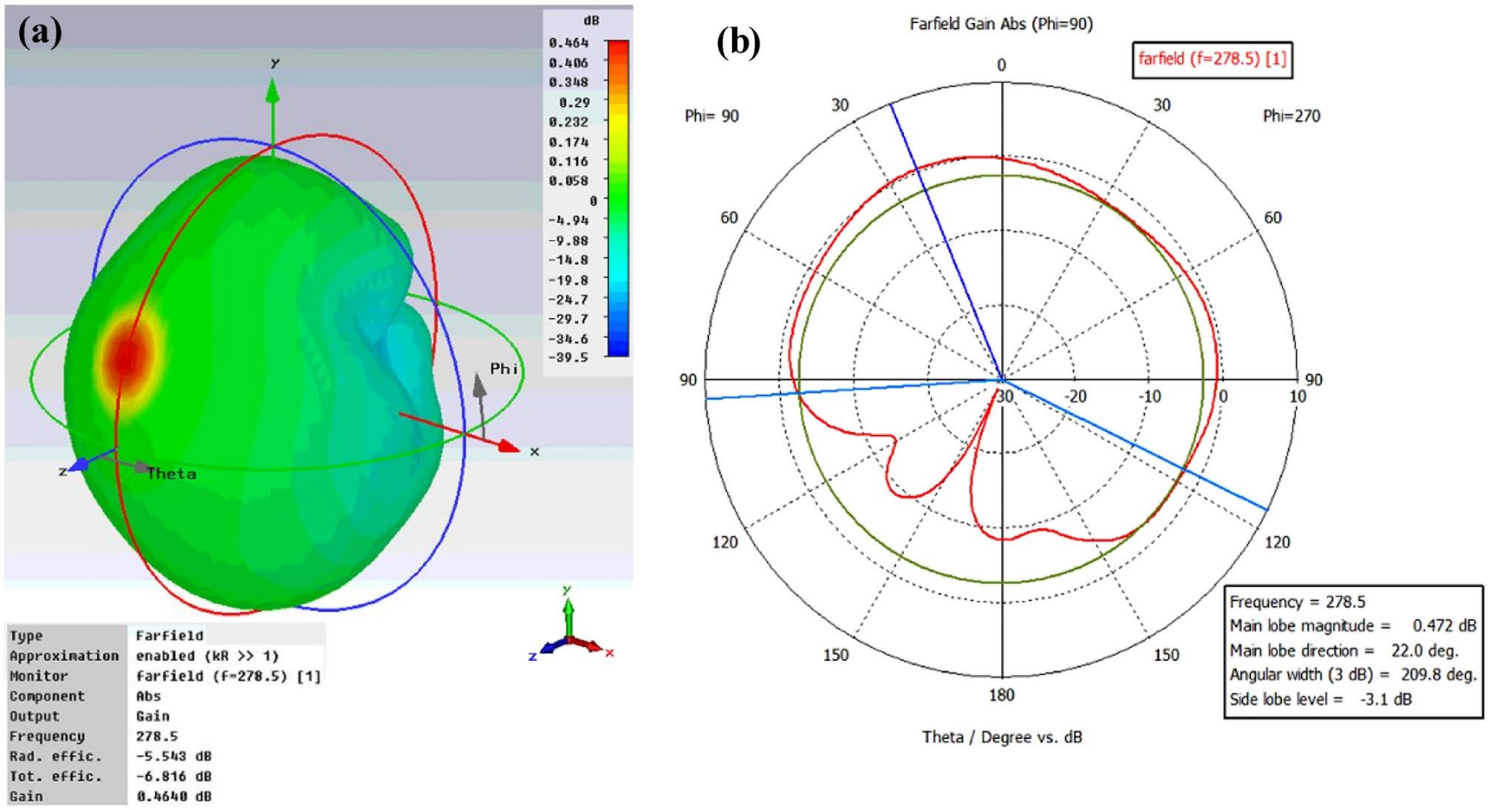
(**a**) 3D and (**b**) polar plot of gain of the proposed unit-cell broadband antenna at 278.5 THz.

The gain and return loss are two important parameters in the design of any antenna. However, there are other parameters such as directivity, half power beam width (HPBW) or 3 dB bandwidth, radiation efficiency, which play a big role and describe the performance of the antenna. It is important to study the radiation pattern of the antenna to know how directional the radiation pattern is or how the single unit cell absorbs EM energy as a function of incident angle. Figure 8a,b illustrate the 3D and polar plot, respectively, of farfield directivity of the proposed antenna at 278.5 THz. The obtained numerical result highlights that the radiation patterns exhibit a maximum directivity value of 6.01 dBi. The half-beam power (HPBW) or 3 dB bandwidth of the antenna at 278.5 THz is 209.8°, which is large enough to assume the antenna is omnidirectional. The high directivity and beam width of the unit cell antenna at 278.5 THz is due to symmetric flower petals geometry. In addition, there are no nulls and lobes present on the pattern results in omnidirectional radiation pattern. Therefore, we can draw a conclusion that the absence of lobes and nulls on the pattern maximize the directivity results in the better performance of the antenna. The gain of the antenna, which is a measure of the maximum effectiveness with which the antenna can radiate or absorb the power delivered to/received by it from the external power or radiation source. More specifically, it is defined as the maximum radiation intensity produced/absorbed by the antenna compared to that given by a lossless isotropic antenna supplied with or received by the same amount of power. The 3D and polar plot of gain of the unit cell antenna shown in Fig. 9a,b, show that the proposed structure provides the optimal gain of 0.464 dB and broader 3 dB bandwidth of 209.8°. The gain of 0.464 dB means that 1.112 times the amount of effective power can be received by the antenna than from an isotropic antenna. The performance results of the proposed unit cell tapered antenna are summarized in Table 7.

**Table 7:** The performance parameters of the proposed unit cell Antenna.

Average absorption ($\theta ={0}^{o}$)	Absorption bandwidth	Angular stability				Directivity	Gain	HPBW
		Polarization ($\theta ={0}^{o},\mathrm{\phi varies}$)		Incident angle ($\theta \mathrm{varies}$)				
84.5%	775 THz	Insensitive	Average absorption	Insensitive	Average absorption	6.01 dBi	0.464 dB	209.8^o^
		Up to 90° (TE)	83.91%	Up to 45^o^ (TE)	82.7%			
		Up to 90° (TM)	83.92%	Up to 45^o^ (TM)	83.5%			

## Conclusions and future work

In this article, a novel UWB tapered flower petal antenna operating in the IR to UV–visible region has been proposed and numerically investigated its performances. The numerical results reveal that the antenna has a wide range absorption capability within the operating frequency bands. The average absorption of 84.5% is obtained by the unit cell antenna in the frequency range from 25 to 800 THz under normal incidence of plane waves. Such high and wideband absorption was achieved through strong electromagnetic coupling, SPPs, plasmonic behavior of the top metal, and overlapping of the consecutive resonance frequencies. The absorption performance remains nearly unchanged under oblique incidence of plane wave up to 45° for both TE and TM mode polarization. The antenna is also polarization insensitive up to 90° for both TE and TM modes under normal incidence of the plane wave. The proposed unit cell antenna also exhibits high directivity and gain that helps to capture EM radiation efficiently from several directions. The numerical findings indicate the technological potential of the proposed antenna design to be utilized in DC power supply modules of low power electronic devices, bolometric sensing, camouflaging, solar power generation, THz imaging, and spectral imaging. We may utilize this antenna to transfer broad bandwidth signals in THz band from base stations as a network backhaul. The tapered antenna design offers the advantage of simplicity, and therefore flexibility in in engineering natural materials for UWB absorption. Furthermore, the design aspect unfolds the new direction for sustainable energy harvesting research in the high frequency regimes (above RF) since the antenna shows UWB absorption in the IR, visible, and part of UV–visible bands. In the near future, we have plan to harvest such high frequency EM waves by using our proposed antenna without any diode based rectification technique.

## Data Availability

The datasets generated during and/or analyzed during the current study are available from the corresponding author on reasonable request.
